# Supplementary material for: Transmission of tauopathy strains is independent of their isoform composition
Source: Nat Commun. 2020 Jan 7;11:7. doi: 10.1038/s41467-019-13787-x (PMC6946697; doi:10.1038/s41467-019-13787-x)
Supplement: Supplementary file 1 — Supplementary Information [file 41467_2019_13787_MOESM1_ESM.pdf]

Supplementary Information

Transmission of Tauopathy Strains is Independent of Their Isoform Composition

He et al.

Contents:

Supplementary Figure 1-9

Supplementary Table 1-3

Supplementary Fig. 1

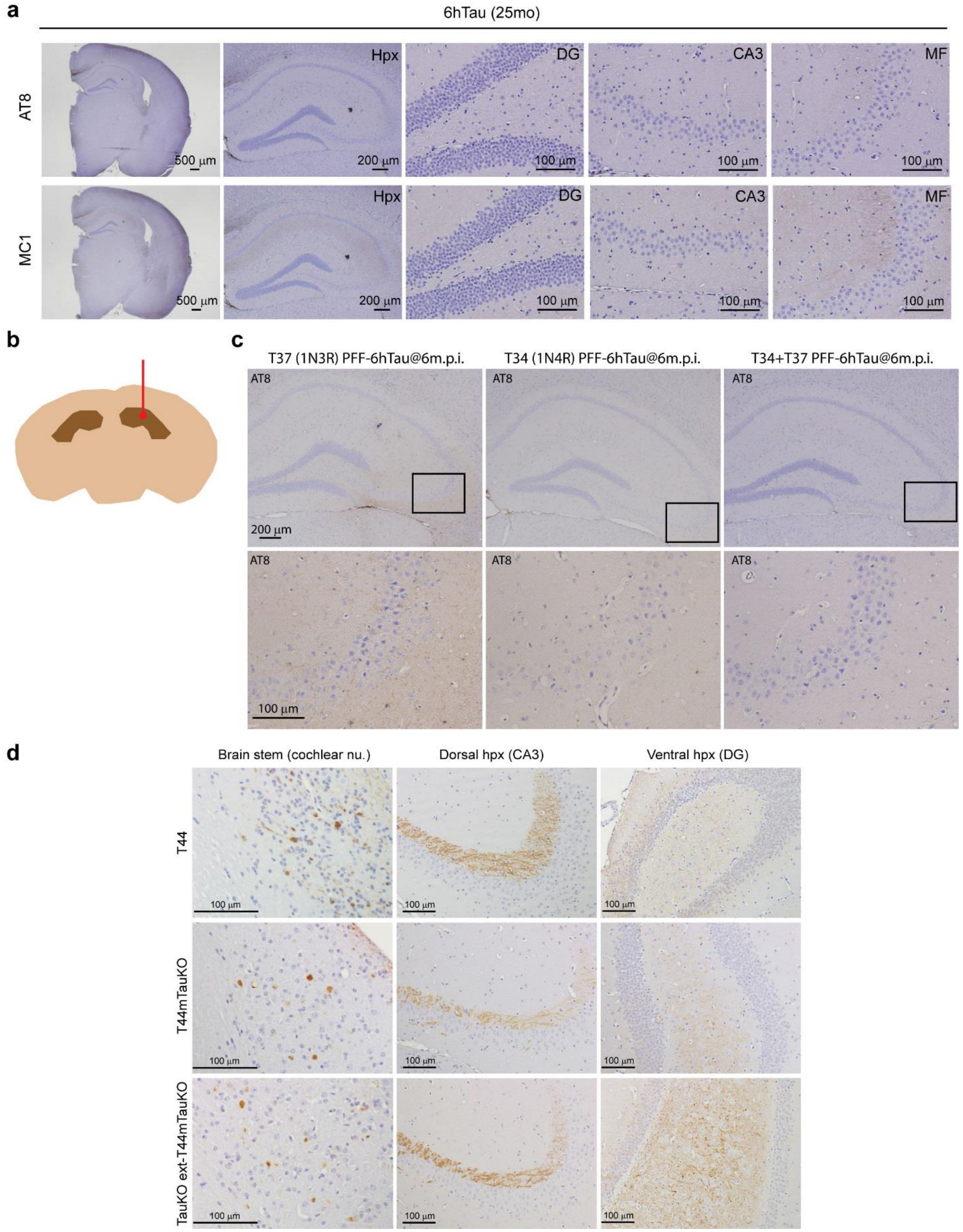

Supplementary Figure 1: Further characterize 6hTau and T44mTauKO mice.

a, Representative IHC staining with anti-hyperphosphorylated tau antibody AT8 and anti-misfolded tau antibody MC1 in the brains of 25 month old 6hTau mice. Note the lack of staining with these pathological tau selective antibodies. **b,** Stereotaxic injection paradigm for preformed fibril (PFF) tau injections into the dorsal hippocampus of the 6hTau mice. **c,** representative IHC staining with AT8 on the 6hTau mice injected with 5 μg PFFs consisting of 3R tau (1N3R, T37) or 4R tau (1N4R, T34) or both 3R and 4R tau isoforms. Mice received the PFF injections at the age of 20-23 months, but no tau pathologies were observed in those mice up to 6 m.p.i. The lower panels are the higher magnification images of the areas in the rectangles in the upper panels. **d**, representative IHC staining with AT8 on the T44 (12 months of age, upper panels), T44mTauKO (8 months of age, middle panels) and T44mTauKO mice injected with brain lysate extracted from mTauKO mice (8 months of age, lower panels). The spontaneously formed granule-like tau pathologies were detected in all three mouse lines, but only restricted to the cochlear nucleus of brain stem. AT8-positive NFTs were barely detectable in other brain regions such as hippocampus. Hpx, hippocampus; DG, dentate gyrus; CA3, cornu ammonis 3; MF, mossy fiber, nu., nucleus.

Supplementary Fig. 2

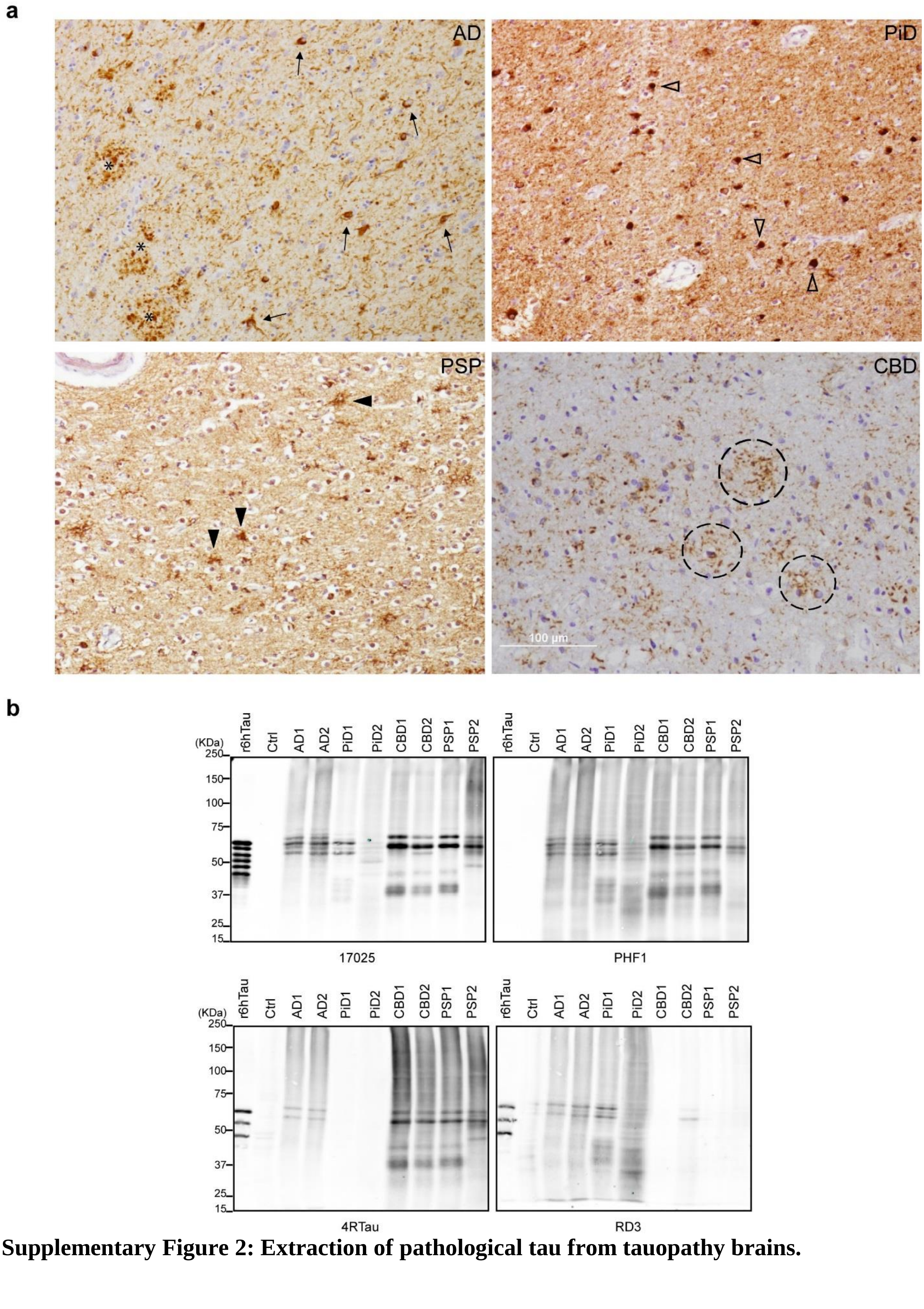

Supplementary Figure 2: Extraction of pathological tau from tauopathy brains.

a, representative IHC performed with anti-hyperphosphorylated tau MAb PHF-1 from the frontal cortices of four human tauopathy brains chosen for tau strain extraction. AD brains showed typical neurofibrillary tangles and neuritic plaques. PiD brains showed typical Pick bodies. In addition to neuronal tau pathologies, PSP brains showed obvious tufted astrocytic tau pathologies, while the CBD brains showed typical astrocytic plaque tau pathologies. Black arrows indicate neurofibrillary tangles and asterisks indicate neuritic plaques in AD brains; open arrowheads indicate Pick's bodies in PiD brains; solid arrowheads indicate tufted astrocytic tau pathologies in PSP brains; dashed circles indicate astrocytic plaques tau pathologies in CBD brains. **b,** different tau strains were extracted from the frontal cortex of tauopathy brains and probed with MAb PHF-1 and rabbit anti-tau antibody 17025. The 3R and 4R tau isoforms were detected by 3R isoform-specific MAb RD3 and 4R isoform-specific polyclonal antibody 4RTau. A control brain without tauopathy was similarly extracted. Recombinant human 6 tau isoforms (r6hTau) were loaded as a standard on the left.

Supplementary Fig. 3

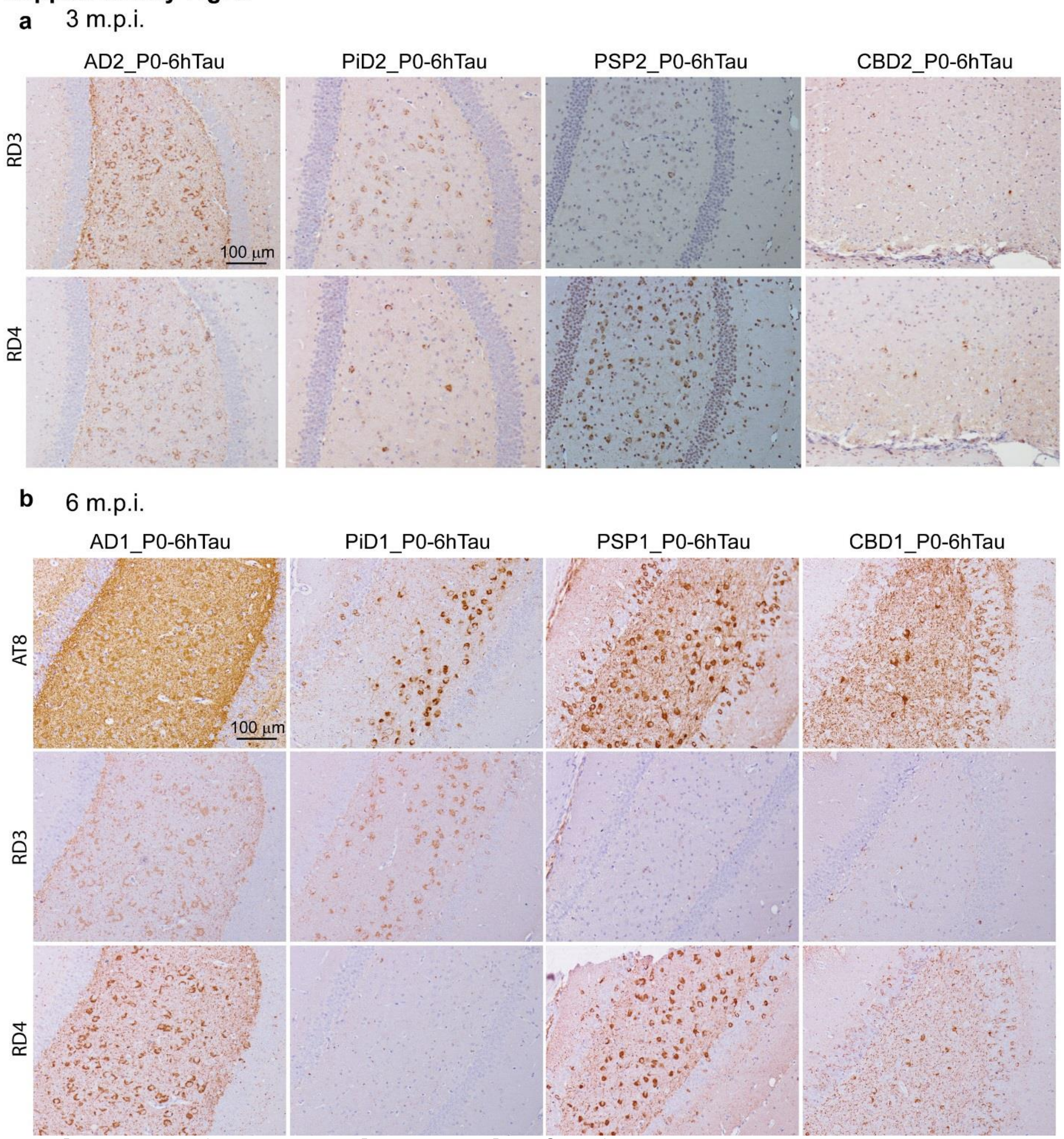

Supplementary Figure 3: Supplementary data for Figure 2.

a, representative IHC staining with tau isoform-specific MAb RD3 and RD4 on 6hTau mice injected with tau strains from a second case of AD (AD2), PiD (PiD2), PSP (PSP2) and CBD (CBD2) at 3 m.p.i.. n=3 for each group. **b,** representative IHC staining with tau MAbs AT8, RD3 and RD4 on 6hTau mice injected with tau strains extracted from the first tauopathy cases (AD1, PiD1, PSP1, CBD1) at 6 m.p.i..

Supplementary Fig. 4

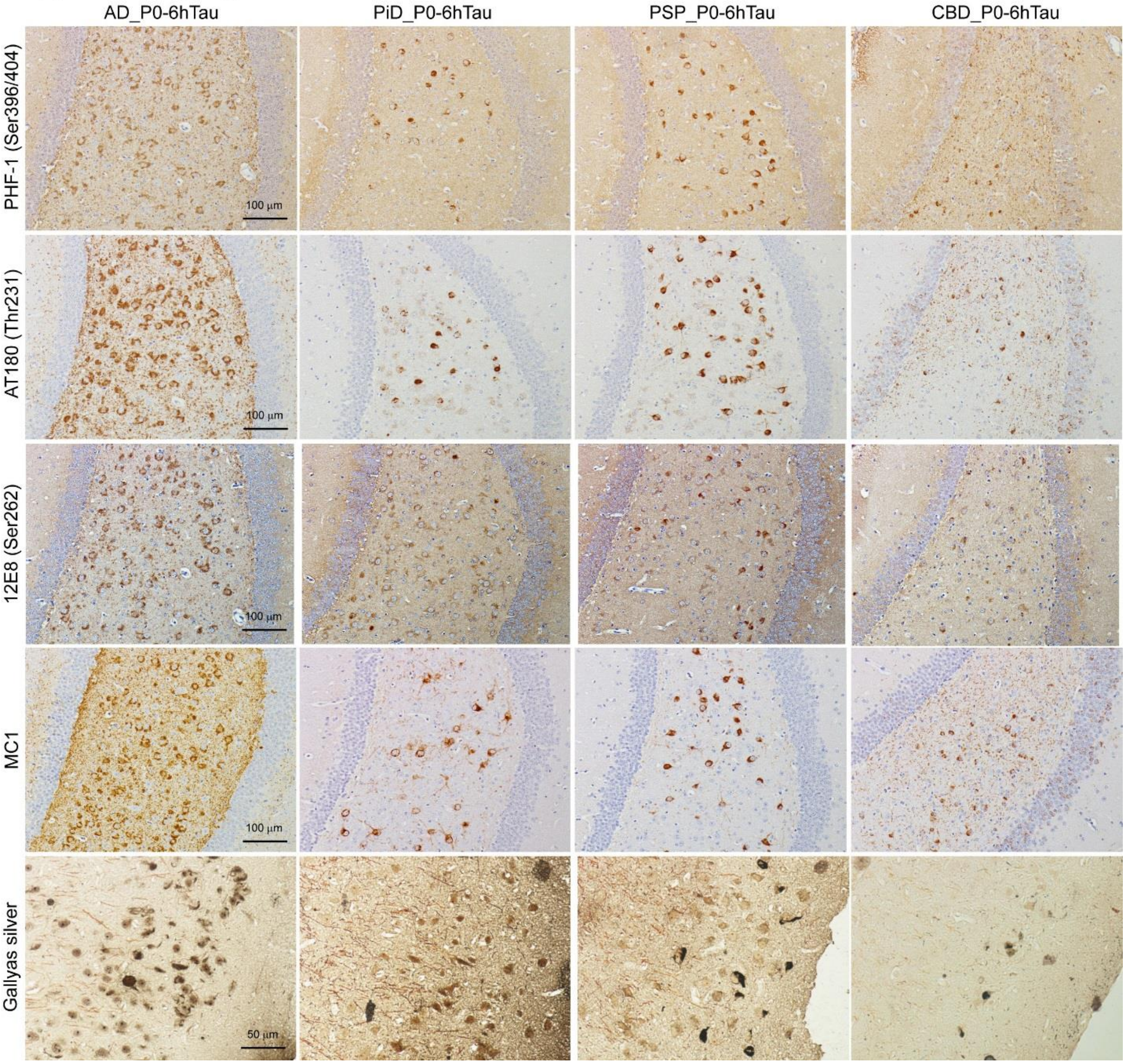

Supplementary Figure 4: Characterization of induced tau pathologies in 6hTau mice.

Tau pathologies induced in 6hTau mice with distinct strain_P0-tau at 3 m.p.i were detected with hyperphosphorylated tau antibodies PHF-1 (Ser396/404), AT180 (Thr231), 12E8 (Ser262) and conformation-dependent antibody MC1. Gallyas silver staining-positive tau pathologies were detected in AD_P0-, PSP_P0-, CBD_P0-6hTau mice at 6 m.p.i., and predominantly enriched in entorhinal cortex, instead of hippocampus, where abundant hyperphosphorylated tau pathologies were detected. The Pick-tau induced tau pathologies were positive for PHF-1, AT180, 12E8, MC1, but negative for Gallya’s sliver staining.

Supplementary Fig. 5

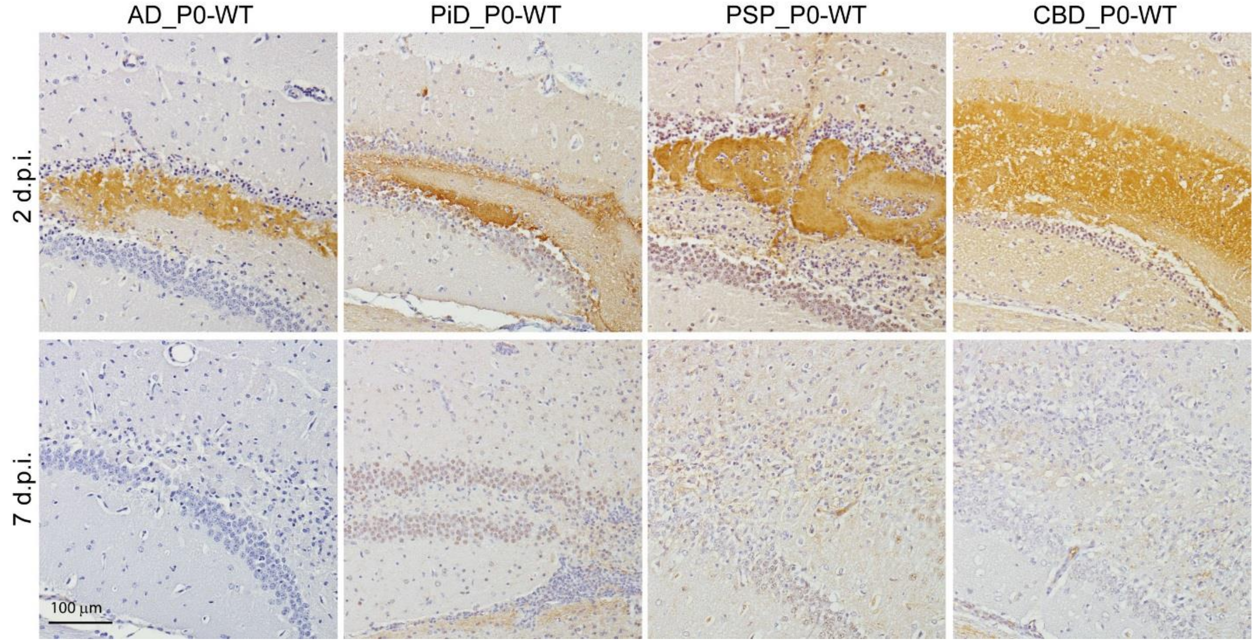

Supplementary Figure 5: Retention of injected human tau materials *in vivo*.

1 μg distinct strain_P0-tau were unilaterally injected into the hippocampus of WT mice, and detected with human tau specific antibody HT-7 at 2 and 7 d.p.i.. Representative IHC staining shows the injection sites in the hippocampal dentate gyrus of each mouse. Two mice per group were injected, and consistently showed similar staining patterns. HT-7 staining were barely detectable at 7 d.p.i. in each tau strain injected WT mice.

Supplementary Fig. 6

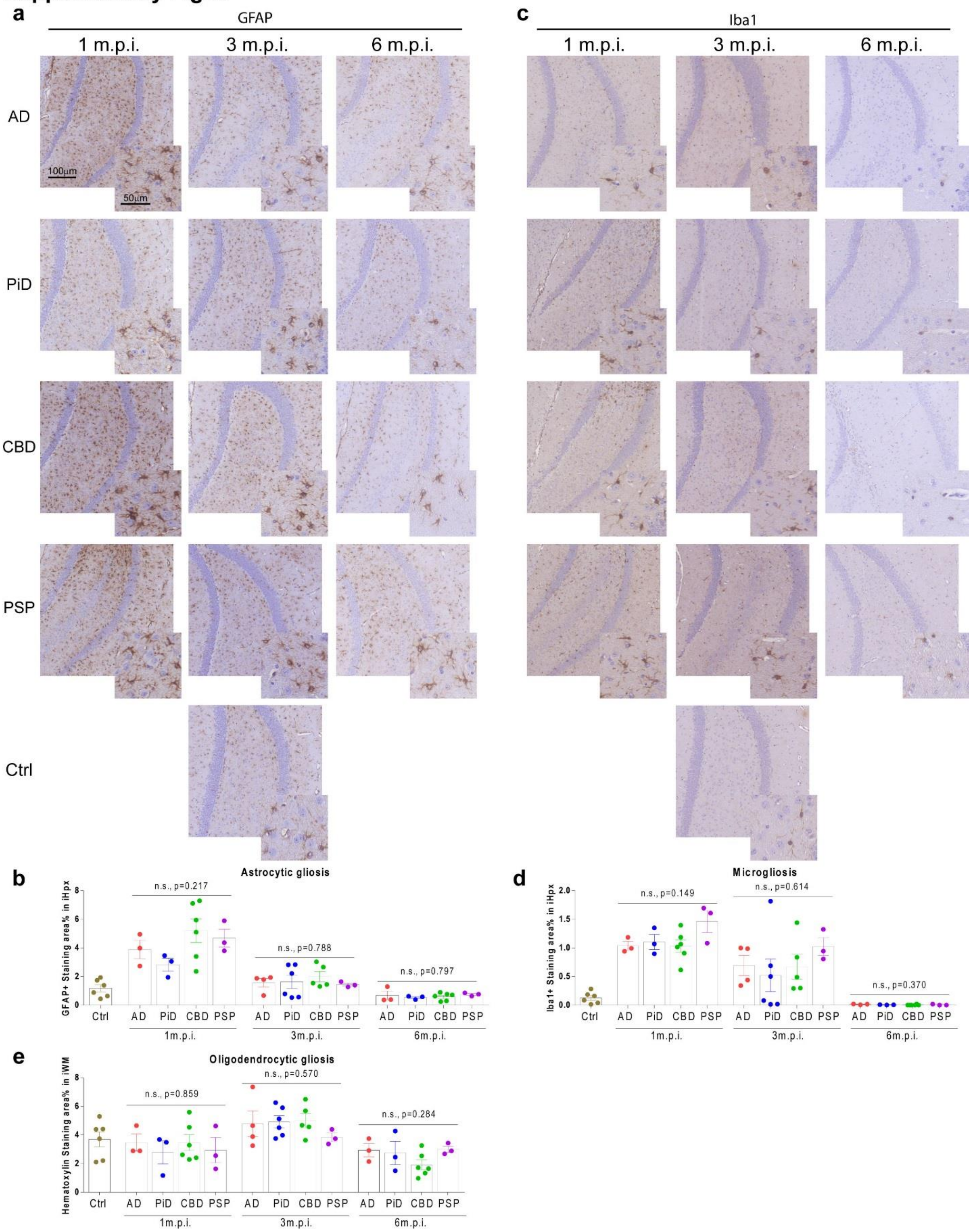

Supplementary Figure 6: Gliosis in 6hTau mice injected with distinct tau strains.

Representative IHC staining with **a**, astrocyte marker GFAP and **c**, microglia marker Iba1 on brain

sections from 6hTau mice injected with distinct tau strain_P0 at 1, 3 and 6 m.p.i. with the same dose 1 μg/site. Inserts are images with higher magnifications. Control slices were from 6hTau mice injected with normal control brain lysate at 3 m.p.i.. **b**, quantification of astrocytes (GFAP) in **a** or **d**, quantification of microglia (Iba1) in **c**. Oligodendrocytic gliosis was examined by measuring the Hematoxyline-labeled nucleus in white matter (WM) regions: corpus collosum and fimbria, as the cells in these white matter tracks are almost exclusive oligodendrocytes. One-way ANOVA tests were performed. At each time point, no significant differences in gliosis were detected. Among multiple time points, the astrocytic and microglial gliosis were generally decreased along with increasing inoculation time, suggesting the higher gliosis detected at 1 m.p.i. was due to mechanical injection injuries rather than the response to the injected tau strains. Note very limited microglial signaling were detected in brain sections 6 m.p.i., probably because most of microglia were at resting state at this time point. The thin neurites of microglia are usually hard to be detected in thin brain sections like our 6 μm ones. Quantifications were from ipsilateral brain side (i) of each mouse. Data are presented as mean ± s.e.m.. n=3-6 mice per group were quantified, each dot represented a mouse.

Supplementary Fig. 7

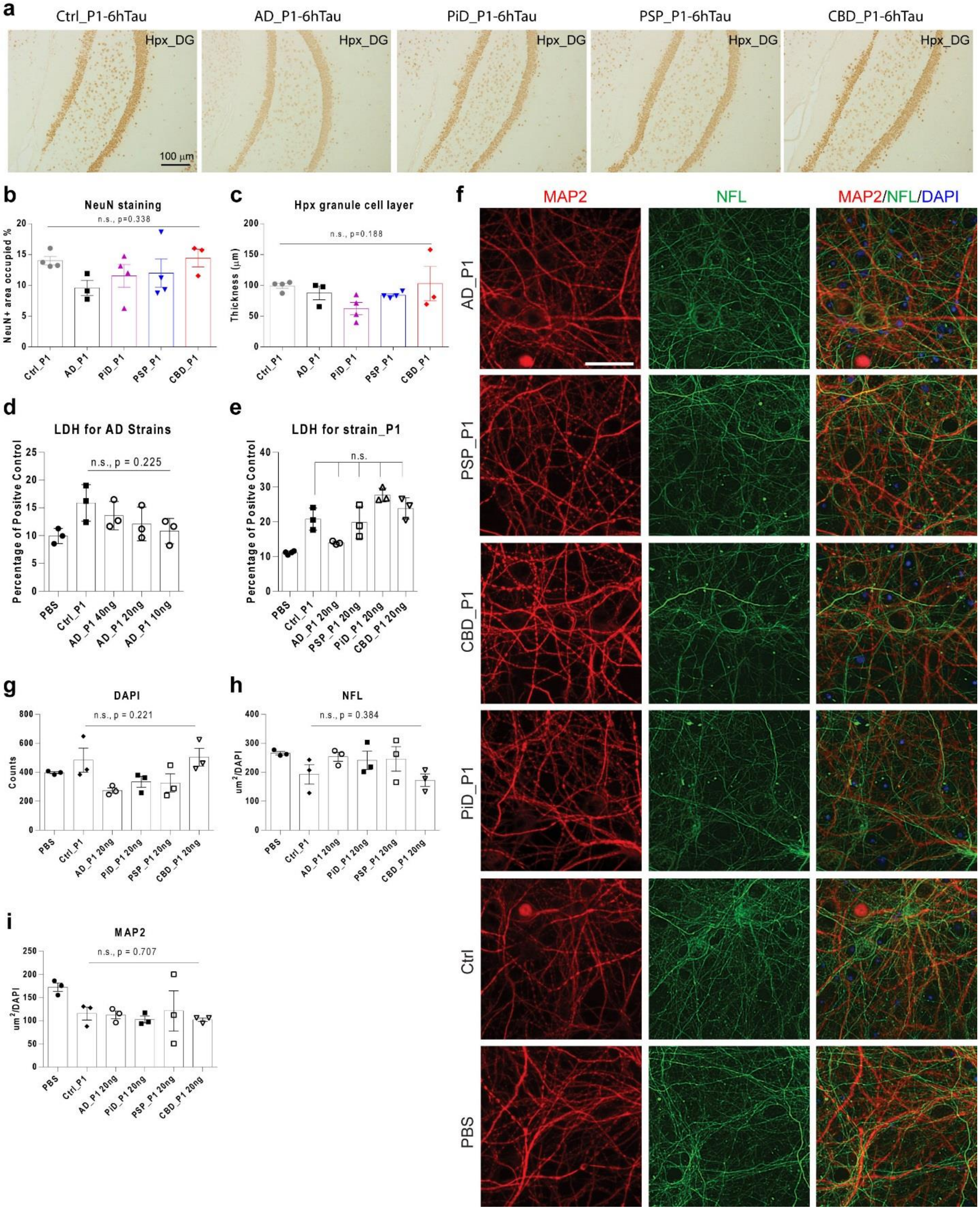

Supplementary Figure 7: Neurotoxicity of strain_P1 *in vivo* and *in vitro*.

a, representative IHC staining with NeuN antibody on brain sections from 6hTau mice injected

with mouse brain lysates containing equal amount of strain_P1 tau (0.2 μg/site). Control mice were injected with brain lysates from non-injected control mouse lysates. The quantification of NeuN were performed on ipsilateral ventral hippocampus, where most of the induced pathologies were enriched. **b**, quantification of total NeuN staining in **a** and **c**, the average thickness of hippocampal DG. One-way ANOVA were performed. **d**, LDH assays of primary cultured neurons treated with different concentration of AD_P1 and **e**, treatment with same dose of different strain_P1 in mouse primary neurons. **f**, representative ICC staining with dendritic (MAP2), axonal (neurofilament light chain, NFL) and nucleus (DAPI) marker in mouse neurons treated with different strain_P1 for two weeks. Ctrl was mouse brain lysate extracted from 6hTau mouse brain injected with 6htau mouse brain lysate to control for the highest total protein level among samples. Scale bar = 50 μm. Quantification of **g,** DAPI; **h**, NFL and **i**, MAP2 immunostaining. DAPI was shown as counts. NFL and MAP2 areas were normalized to DAPI counts. Data are presented as mean ± s.e.m. and each dot represents a batch repeat. One-way ANOVA with multiple comparisons were performed. Significant differences were considered when $p \leq 0.05$.

Supplementary Fig. 8

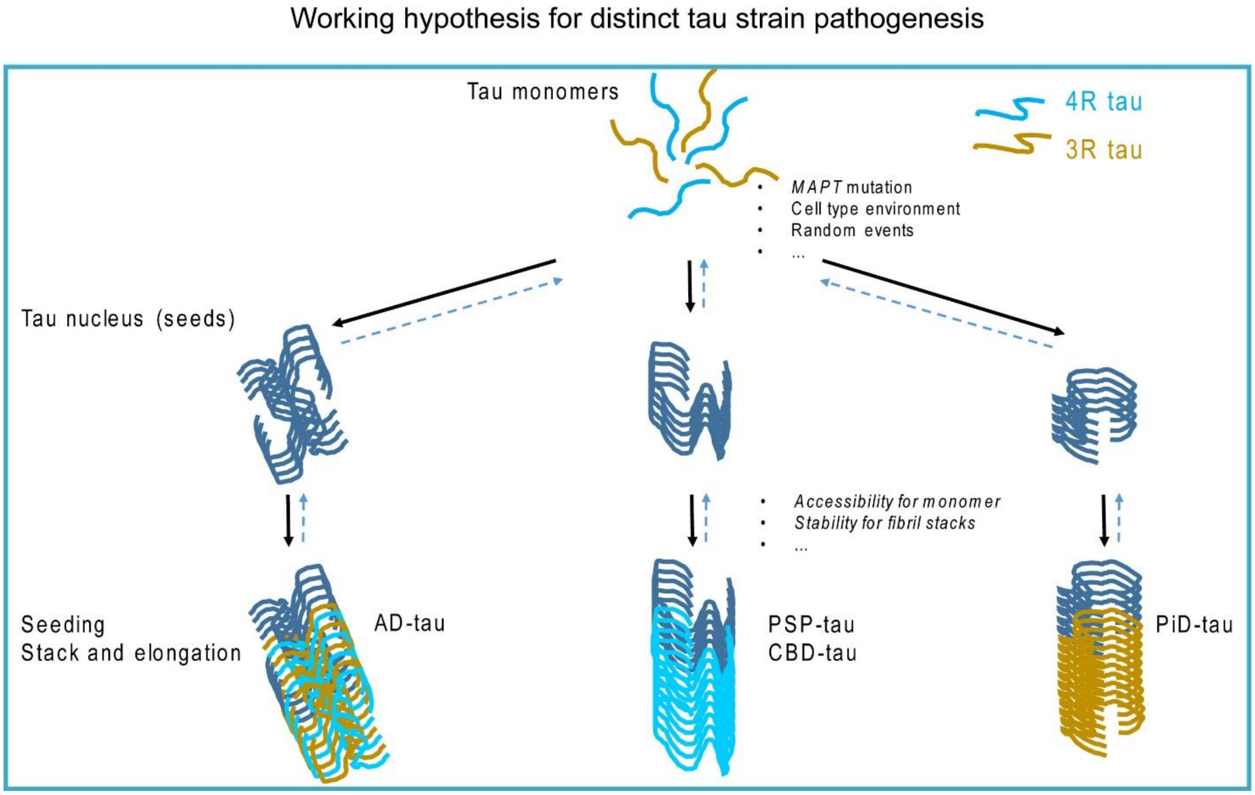

Supplementary Figure 8: Working hypothesis for distinct tau strain pathogenesis.

3R or 4R tau monomers were respectively labelled as light blue or brown curve bars. The initial seeds with different conformations are indicated in dark blue. The initial tau pathogenesis could be divided into two phases: the initial nucleus seed formation phase followed by amyloidogenic amplification (seeding) phase, where the endogenous monomeric tau isoforms are templated by and stacked to the misfolded seeds, thereby elongating the fibrils. Differences in cell type environment, random events like somatic mutations, may contribute to the initial formation of tau seeds. The conformational differences among the initial tau strain seeds would lead to differential accessibility for different isoform monomers, resulting in different isoform recruitment. For example, the initial AD-tau seeds enable the recruitment of both 3R and 4R tau monomers to form the fibrillization core. During the amplification phase, the fibrillization core containing both 3R and 4R tau provides a platform for the stacking of either 3R, 4R or a mixture of 3R and 4R tau into polymorphs. However, for CBD or PSP strains, the initial seeds are formed with a preferred accessibility for 4R tau isoforms to be recruited, and later in the amplifying process are more compatible for 4R tau isoforms to stack into the fibrils. For the PiD strain, its core region appears to be quite different from the AD strain, and prefer to recruit 3R tau isoforms to stack into the fibrils. Once such elongation started, stacking of non-pairing isoform monomers would cause the instability of the assembly, leading to the breakdown of filament structures. As a result, distinct strains show isoform-specific seeding pattern.

Supplementary Fig. 9

Fig. 1a

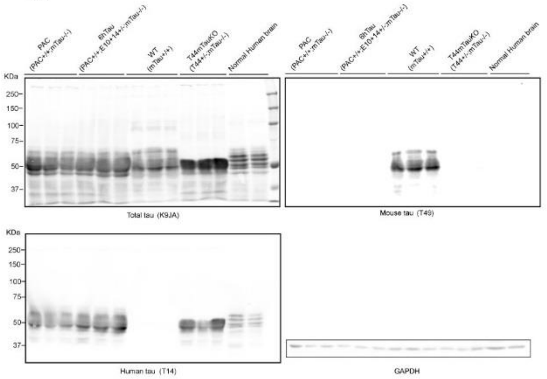

Fig. 1c

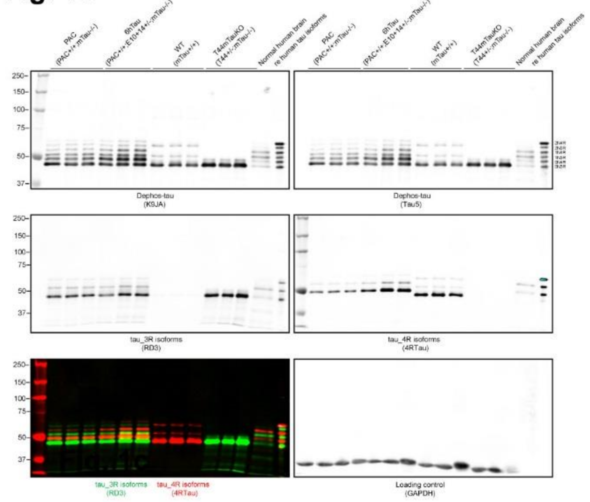

Fig. 1e

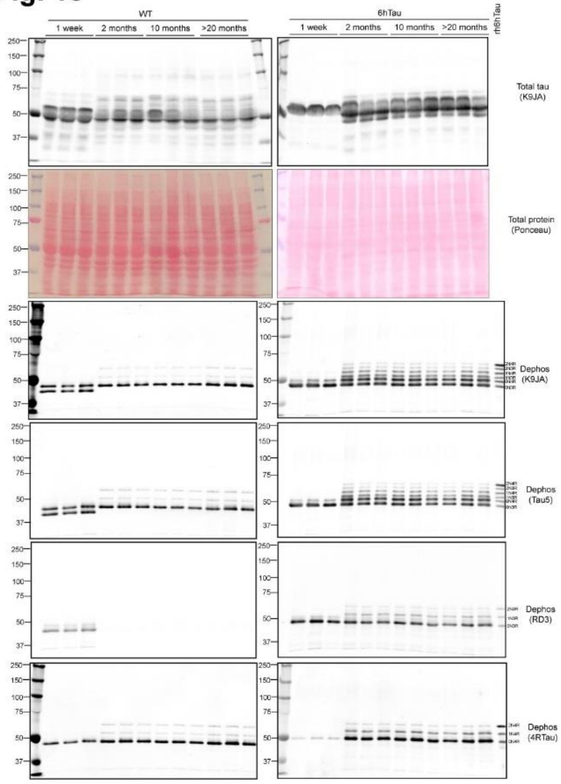

Fig. 1j

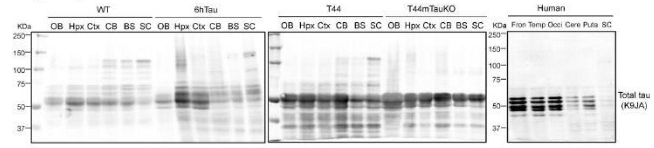

Fig. 1l

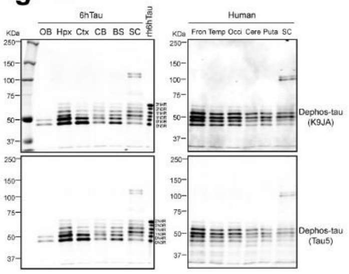

Fig. 4b

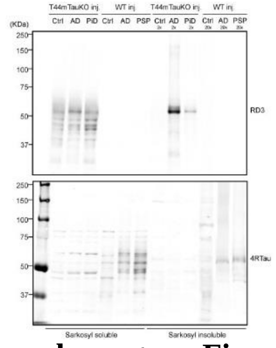

Fig. 4e

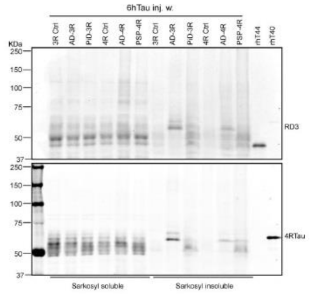

Supplementary Figure 9: Uncropped blots shown in main figures.

Supplementary Table 1. Demographics of human cases used in this study

Case No.	Neuropathological Diagnosis	Gender	Age at death	PMI (hr)	Disease duration (year)
1	Normal	M	62	16	None
2	Normal	M	59	17	None
3	Normal	M	73	17	None
4	Normal	M	66	20	None
5	AD	F	68	9	8
6	AD	F	59	14	9
7	PiD	M	71	4	17
8	PiD	M	74	24	6
9	CBD	F	56	15	6
10	CBD	M	52	8	4
11	PSP	F	63	6.5	5
12	PSP	M	89	7.5	5

Supplementary Table 2. Antibodies used in these studies

Antibody Name	Specificity	Host Species	Dilutions	Source
AT8	p-tau (phosphorylated at Ser202 and Thr 205)	mouse monoclonal, IgG1	1:1000 (WB); 1:10000 (IHC); 1:2000 (IF)	Thermo Scientific, MN1020
PHF-1	p-tau (phosphorylated at Ser396 and Ser404)	mouse monoclonal, IgG1	1:1000 (WB)	Gift from Dr. Peter Davies
AT180	p-tau (phosphorylated at Thr281)	mouse monoclonal, IgG1	1:1000 (IHC)	Thermo Scientific, MN1040
12E8	p-tau (phosphorylated at pSer262/pSer356	mouse monoclonal, IgG1	1:5000 (IHC)	Prothena Biosciences
MC1	tau in pathological conformation	mouse monoclonal, IgG1	1:7000 (IHC)	Gift from Dr. Peter Davies
T49	Mouse tau	mouse monoclonal	1:1000 (WB)	In-house
T14	Human tau	mouse monoclonal	1:3000 (WB)	In-house
HT7	Human tau (aa 159-163)	mouse monoclonal	1:500 (IHC)	Thermo Scientific, MN1000
Biotinylated HT7	tau (aa 159-163)	mouse monoclonal	62.5 ng/ml as reporting antibody in tau ELISA together with BT2	Thermo Scientific, MN1000B
17025	Recombinant human tau	rabbit polyclonal	1:5000 (WB)	In-house
K9JA	tau (aa 243-441)	rabbit polyclonal	1:5000 (WB)	Dako, A0024
Anti-4R tau	4R Tau	rabbit polyclonal	1:5000 (WB)	Cosmo Bio Co., TIP-4RT-P01
RD4	4R Tau	mouse monoclonal	1:10000 (IHC)	Millipore, 05-804
RD3	3R Tau	mouse monoclonal	1:10000 (IHC)	Millipore, 05-803
GT38	AD 3R and 4R mix	mouse monoclonal	1:1000 (IHC)	In-house
R2295M	Mouse tau	rabbit polyclonal	1:2000 (ICC)	In-house
NFL	Neurofilament light chain	rabbit polyclonal	1:1000 (ICC)	In-house
MAP2	Microtubule associate protein 2	chicken polyclonal	1:2000 (ICC)	Millipore, AB5543
Biotinylated	tau (aa 194-198)	mouse	31.25 ng/ml as reporting	Thermo

BT2		monoclonal	antibody in tau ELISA together with HT7	Scientific, MN1010B
Oligo2	Oligo2	rabbit polyclonal	1:250 (IF)	Millipore, AB9610
GFAP	GFAP	rabbit polyclonal	1:3000 (IF), 1:20000 (IHC)	Dako, Z0334
Iba 1	Iba 1	rabbit polyclonal	1:1000 (IHC)	Wako, 019-19741
NeuN	NeuN	mouse monoclonal	1:500 (IHC)	Millipore, MAB377
9027	α-syn (aa 130-140)	mouse monoclonal	3.3 µg/ml as capture antibody for α-syn ELISA	In-house
MJF-R1	α-syn (aa 118-123)	rabbit monoclonal	0.5425 µg/ml as reporting antibody for α-syn ELISA	Abcam (ab138501)
Ban 50	Aβ (aa1-10)	mouse monoclonal	7.5 µg/ml as capture antibody for Aβ ELISA	In-house
HRP-labeled BA27	Aβ1-40	mouse monoclonal	1:1000 as reporting antibody for Aβ1-40 ELISA	Takeda Pharmaceutical
HRP-labeled BC05	Aβ1-42	mouse monoclonal	1:500 as reporting antibody for Aβ1-42 ELISA	Takeda Pharmaceutical

Abbreviations: WB - western blotting; IHC -IHC; IF – immunofluorescence; ICC-immunocytochemistry.

Supplementary Table 3. Statistical analysis details

Fig. #	Compare (group size n=)	Statistical method	P value	t value	F value	Degrees of freedom
Fig. 2h	AD_P0-6hTau RD3 vs. RD4	Multiple t-test	0.424			8
	PiD_P0-6hTau RD3 vs. RD4	Multiple t-test	<0.0001			10
	PSP_P0-6hTau RD3 vs. RD4	Multiple t-test	0.027			4
	CBD_P0-6hTau RD3 vs. RD4	Multiple t-test	0.003			10
Fig. 2i	AD_P1-6hTau RD3 vs. RD4	Multiple t-test	0.616			8
	PiD_P1-6hTau RD3 vs. RD4	Multiple t-test	0.023			4
	PSP_P1-6hTau RD3 vs. RD4	Multiple t-test	<0.0001			4
	CBD_P1-6hTau RD3 vs. RD4	Multiple t-test	0.026			4
Fig. 2j	AD_P1-6hTau RD3 vs. RD4	Multiple t-test	>0.999			4
	PSP_P1-6hTau RD3 vs. RD4	Multiple t-test	0.016			4
	CBD_P1-6hTau RD3 vs. RD4	Multiple t-test	0.374			4
Fig. 3e	AT8+ neuron number in T44mTauKO mice injected with different tau strains at 3m.p.i.	One-way ANOVA	<0.0001		F(3, 8)=3098	11
	AD-T44TKO vs. PSP-T44TKO	Sidak's multiple comparisons test	<0.0001	83.42		8
	AD-T44TKO vs. CBD-T44TKO	Sidak's multiple comparisons test	<0.0001	83.46		8
	PiD-T44TKO vs. PSP-T44TKO	Sidak's multiple comparisons test	<0.0001	25.17		8
	PiD-T44TKO vs. CBD-T44TKO	Sidak's multiple comparisons test	<0.0001	25.21		8
	AT8+ neuron number in WT mice injected with different tau strains at 3m.p.i.	One-way ANOVA	<0.0001		F (3, 17) = 27.05	20
	PiD-WT vs. PSP-WT	Sidak's multiple comparisons test	<0.0001	7.696		17

Fig. 3f	AT8+ neuron number in T44mTauKO mice injected with different tau strains at 6m.p.i.	One-way ANOVA	<0.0001		F (3, 8) = 403.0	11
	AD-T44TKO vs. PSP-T44TKO	Sidak's multiple comparisons test	<0.0001	29.95		8
	AD-T44TKO vs. CBD-T44TKO	Sidak's multiple comparisons test	<0.0001	29.99		8
	PiD-T44TKO vs. PSP-T44TKO	Sidak's multiple comparisons test	<0.0001	12.76		8
	PiD-T44TKO vs. CBD-T44TKO	Sidak's multiple comparisons test	<0.0001	12.80		8
	AT8+ neuron number in WT mice injected with different tau strains at 6m.p.i.	One-way ANOVA	0.0001		F (3, 13) = 16.17	16
	PiD-WT vs. PSP-WT	Sidak's multiple comparisons test	0.0002	5.731		13
	PiD-WT vs. CBD-WT	Sidak's multiple comparisons test	0.0231	3.144		13
Fig. 4f	AD_3R-6hTau 3R vs. 4R	Multiple t-test	0.478			4
	PiD_3R-6hTau 3R vs. 4R	Multiple t-test	0.013			4
	AD_4R-6hTau 3R vs. 4R	Multiple t-test	0.708			4
	PSP_4R-6hTau 3R vs. 4R	Multiple t-test	0.085			4
Fig. 4h	AD_3R-WT vs. PiD_3R-WT	Mann Whitney test, one-tailed	0.05			
Fig. 4i	AD_4R-T44mTauKO vs. PSP_4R-T44mTauKO	Mann Whitney test, one-tailed	0.05			
Fig. 6b	AD_P0 vs. AD_P1	T-test, two-tailed	0.096			
	PiD_P0 vs. PiD_P1	T-test, two-tailed	0.488			
	PSP_P0 vs. PSP_P1	T-test, two-tailed	0.020			
	CBD_P0 vs. CBD_P1	T-test, two-tailed	0.156			
Fig. 8c	AD_P0 vs. AD_P1	T-test, two-tailed	0.423			
	PiD_P0 vs. PiD_P1	T-test, two-tailed	0.061			
	PSP_P0 vs. PSP_P1	T-test, two-tailed	0.517			
	CBD_P0 vs. CBD_P1	T-test, two-tailed	0.306			
Fig. 8d	AD_P0 vs. AD_P1	T-test, two-tailed	0.423			
	PiD_P0 vs. PiD_P1	T-test, two-tailed	0.852			
	PSP_P0 vs. PSP_P1	T-test, two-tailed	0.138			
	CBD_P0 vs. CBD_P1	T-test, two-tailed	0.217			
Fig. 8f	Among different AD dose	One-way ANOVA	<0.0001		F(7, 16)= 54.5	23

	40 ng AD_P0 vs. AD_P1	Tukey's multiple comparisons test	0.072			16
	20 ng AD_P0 vs. AD_P1	Tukey's multiple comparisons test	0.650			16
	10 ng AD_P0 vs. AD_P1	Tukey's multiple comparisons test	0.947			16
Fig. 8g	Among different PSP dose	One-way ANOVA	<0.0001		F(7, 16)= 93.81	23
	40 ng AD_P0 vs. AD_P1	Tukey's multiple comparisons test	0.842			16
	20 ng AD_P0 vs. AD_P1	Tukey's multiple comparisons test	0.708			16
	10 ng AD_P0 vs. AD_P1	Tukey's multiple comparisons test	0.666			16
Sup. Fig. 7e	CBD_P0-6hTau RD3 vs. RD4	Multiple t-test	0.006			10
Sup. Fig. 7f	CBD_P1-6hTau RD3 vs. RD4	Multiple t-test	0.184			4
Sup. Fig. 7h	PSP_4R-6hTau RD3 vs. RD4	Multiple t-test	0.006			4
Fig. 9b	ThioS+/AT8+% cell in 6hTau mice injected with different tau strains at 3 m.p.i..	One-way ANOVA	<0.0001		F (3, 16) = 78.60	19
	3m.p.-AD-6hTau vs. 3m.p.-PiD-6hTau	Sidak's multiple comparisons test	<0.0001	13.03		16
	3m.p.-AD-6hTau vs. 3m.p.-PSP-6hTau	Sidak's multiple comparisons test	<0.0001	6.635		16
	3m.p.-AD-6hTau vs. 3m.p.-CBD-6hTau	Sidak's multiple comparisons test	<0.0001	13.81		16
	3m.p.-PiD-6hTau vs. 3m.p.-PSP-6hTau	Sidak's multiple comparisons test	<0.01	4.309		16
	3m.p.-PSP-6hTau vs. 3m.p.-CBD-6hTau	Sidak's multiple comparisons test	<0.0001	4.973		16
	ThioS+/AT8+% cell in 6hTau mice injected with different tau strains at 6 m.p.i..	One-way ANOVA	<0.0001		F (3, 11) = 24.12	14
	6m.p.-AD-6hTau vs. 6m.p.-PiD-6hTau	Sidak's multiple comparisons test		3.678		11
	6m.p.-AD-6hTau vs. 6m.p.-CBD-6hTau	Sidak's multiple comparisons test		7.739		11
	6m.p.-PiD-6hTau vs. 6m.p.-CBD-6hTau	Sidak's multiple comparisons test		3.492		11

	6m.p.-PSP-6hTau vs. 6m.p.-CBD-6hTau	Sidak's multiple comparisons test		5.909		11
Fig. 9e	GT38+/AT8+% cell in 6hTau mice injected with different tau strains	One-way ANOVA	<0.0001		F (3, 23) = 13.80	26
Sup. Fig. 9b	PSP_P0-6hTau RD3 vs. RD4	Multiple t-test	0.015			4
	CBD_P0-6hTau RD3 vs. RD4	Multiple t-test	0.004			10
Sup. Fig. 9c	PSP_P1-6hTau RD3 vs. RD4	Multiple t-test	0.010			4
	CBD_P1-6hTau RD3 vs. RD4	Multiple t-test	0.026			4
Sup. Fig. 6b	GFAP+ staining% among different strain injected 6hTau at 1 m.p.i.	One-way ANOVA	0.217		F (3, 11) = 1.440	14
	GFAP+ staining% among different strain injected 6hTau at 3 m.p.i.	One-way ANOVA	0.788		F (3, 14) = 0.3528	17
	GFAP+ staining% among different strain injected 6hTau at 6 m.p.i.	One-way ANOVA	0.797		F (3, 11) = 0.3396	14
Sup. Fig. 6d	Iba1+ staining% among different strain injected 6hTau at 1 m.p.i.	One-way ANOVA	0.149		F (3, 11) = 2.170	14
	Iba1+ staining% among different strain injected 6hTau at 3 m.p.i.	One-way ANOVA	0.614		F (3, 14) = 0.6187	17
	Iba1+ staining% among different strain injected 6hTau at 6 m.p.i.	One-way ANOVA	0.370		F (3, 11) = 1.157	14
Sup. Fig. 6e	Hematoxylin+ staining% among different strain injected 6hTau at 1 m.p.i.	One-way ANOVA	0.859		F (3, 11) = 0.2515	14
	Hematoxylin+ staining% among different strain injected 6hTau at 3 m.p.i.	One-way ANOVA	0.570		F (3, 14) = 0.6952	17
	Hematoxylin+ staining% among different strain injected 6hTau at 6 m.p.i.	One-way ANOVA	0.284		F (3, 11) = 1.440	14
Sup. Fig. 7b	NeuN staining area occupied % comparison among strain_P1.	One-way ANOVA	0.338		F(4,13)=1.250	17

Sup. Fig. 7c	Hippocampal DG thickness comparison among strain_P1.	One-way ANOVA	0.188		F(4,13)=1.807	17
Sup. Fig. 7d	LDH for AD strain at different dose comparison	One-way ANOVA	0.225		F(3,8)=1.798	11
Sup. Fig. 7e	LDH for strain_P1 comparison	One-way ANOVA	0.003		F(4,10)=8.558	14
	Ctrl vs. AD_P1	Tukey's multiple comparisons test	0.112			10
	Ctrl vs. PSP_P1	Tukey's multiple comparisons test	0.995			10
	Ctrl vs. PiD_P1	Tukey's multiple comparisons test	0.104			10
	Ctrl vs. CBD_P1	Tukey's multiple comparisons test	0.723			10
Sup. Fig. 7g	DAPI for strain_p1 comparison	One-way ANOVA	0.221		F(2,8)=0.2987	14
Sup. Fig. 7h	NFL for strain_p1 comparison	One-way ANOVA	0.384		F(2,8)=0.373	14
Sup. Fig. 7i	MAP2 for strain_p1 comparison	One-way ANOVA	0.707		F(2,8)=3.303	14
